# The pH Influence on the Water-Splitting Electrocatalytic Activity of Graphite Electrodes Modified with Symmetrically Substituted Metalloporphyrins

**DOI:** 10.3390/nano12213788

**Published:** 2022-10-27

**Authors:** Bogdan-Ovidiu Taranu, Eugenia Fagadar-Cosma

**Affiliations:** 1 National Institute for Research and Development in Electrochemistry and Condensed Matter, Dr. A. Paunescu Podeanu Street No. 144, 300569 Timisoara, Romania; 2 Institute of Chemistry “Coriolan Dragulescu”, Mihai Viteazu Ave. 24, 300223 Timisoara, Romania

**Keywords:** metalloporphyrins, aggregates, electrocatalysis, water splitting, electron microscopy

## Abstract

Hydrogen, considered to be an alternative fuel to traditional fossil fuels, can be generated by splitting water molecules into hydrogen and oxygen via the use of electrical energy, in a process whose efficiency depends directly on the employed catalytic material. The current study takes part in the relentless search for suitable and low-cost catalysts relevant to the water-splitting field by investigating the electrocatalytic properties of the O_2_ and H_2_ evolution reactions (OER and HER) of two metalloporphyrins: Zn(II) 5,10,15,20-tetrakis(4-pyridyl)-porphyrin and Co(II) 5,10,15,20-tetrakis(3-hydroxyphenyl)-porphyrin. The TEM/STEM characterisation of the porphyrin samples obtained using different organic solvents revealed several types of self-assembled aggregates. The HER and OER experiments performed on porphyrin-modified graphite electrodes in media with different pH values revealed the most electrocatalytically active specimens. For the OER, this specimen was the electrode manufactured with one layer of Co-porphyrin applied from dimethylsulfoxide, exhibiting an overpotential of 0.51 V at i = 10 mA/cm^2^ and a Tafel slope of 0.27 V/dec. For the HER, it was the sample obtained by drop casting one layer of Zn-porphyrin from N,N-dimethylformamide that displayed a HER overpotential of 0.52 V at i = −10 mA/cm^2^ and a Tafel slope of 0.15 V/dec.

## 1. Introduction

The continuous and rapid increase in the human population worldwide is directly related to the escalating global energy demand. This demand is currently being addressed by using renewable and non-renewable energy sources, with the caveat that non-renewable fuels contribute substantially to the total energy production [1] despite their negative impact on the environment [2]. Instead, the focus should be on the development of eco-friendly technologies for fuels that could be used to avoid a future energy crisis. Hydrogen is regarded as an alternative to the traditional sources that are environmentally harmful due to their combustion products [3]. While it can be produced with technologies that rely on fossil fuels (such as coal gasification, steam reforming, and catalytic partial oxidation), it can also be obtained by employing solar, wind, wave, and tidal energy [1,4]. The specified eco-friendly energy sources are employed to procure hydrogen by powering the water electrolysis process [5], which is responsible for the decomposition of water molecules into hydrogen and oxygen via an electric current [6]. Once generated, hydrogen can be stored, moved, and either reconverted to electrical energy via fuel cells or used to obtain different fuels [7]. Important progress has been made in the water-splitting domain [8,9,10], and one noticeable aspect of this progress concerns the materials utilized to catalyse the two half-cell reactions involved in water electrolysis, namely, the hydrogen evolution reaction (HER), during which H_2_ is produced at the cathode as a result of water reduction, and the oxygen evolution reaction (OER), responsible for the formation of the O_2_ molecule by anodic water oxidation [11]. The sluggish kinetics of these reactions—especially that of OER, which involves a four-electron transfer and is the main bottleneck in water electrolysis [12]—constitute an important reason for why the water-splitting process is of limited practical use. In order to decrease the HER and OER overpotentials and ensure efficient gas production, many materials with catalytic properties towards the two reactions have been considered [13,14,15]. Noble metal-based catalysts are known for their high catalytic activity and stability, but their scarcity and high cost are serious problems for large scale applications [16].Thus, the focus has currently shifted to low-cost and earth-abundant, non-noble metal-based catalysts, which usually contain transition metals, but can also be metal-free [17,18]. One class of compounds that has been studied in terms of its electrocatalytic properties for water splitting—with promising results—is that which constitutes the extended aromatic macrocycles known as porphyrins [19]. They share a common tetrapyrrolic ring structure, which can theoretically bind almost all metal cations, resulting in metalloporphyrins [20,21].

Metalloporphyrins have been studied as electrocatalysts for water electrolysis since before the 21st century. One example is the study reported in 1985 by Kellett et al. [22], in which a series of cobalt porphyrins were investigated as homogeneous HER catalysts. An example regarding the study of metalated porphyrins as OER catalysts is the one reported by Naruta et al. [23], who used manganese porphyrin dimers. While in the early research macromolecules were mostly employed as homogeneous activators, their use as heterogeneous catalysts has gained ground [24], and in light of promising experimental data they have been extensively investigated as water-splitting catalysts. The progress made in the past decade concerns the identification and detailed understanding of the critical factors responsible for the water-electrocatalytic properties of the porphyrin complexes [19]. It is also worth noting that metalloporphyrins have been successfully used in combination with other materials to manufacture complex electrodes possessing HER and OER electrocatalytic properties, result in part from the synergistic effect of the employed mixture [25].

The present work describes the evaluation of the OER and HER electrocatalytic properties of two metalloporphyrins: Zn(II) 5,10,15,20-tetrakis(4-pyridyl)-porphyrin (ZnP) and Co(II) 5,10,15,20-tetrakis(3-hydroxyphenyl)-porphyrin (CoP). These metalated structures have been the subject of several articles published by Fagadar et al. [26,27,28,29,30,31,32,33,34,35], but the researchers did not investigate their applicative potential in the water-splitting field. As for the studies reported by other research groups, a literature survey revealed the extent to which the two porphyrin derivatives were analysed in terms of their HER and OER properties. Three published works have been identified for ZnP. Thus, Gianoudis et al. [36] reported a comparison between a series of zinc and tin metalloporphyrins (including ZnP) employed as photosensitizers for photochemical H_2_ evolution using cobaloxime complexes as molecular catalysts. In addition, Hasobe et al. [37] developed a visible light-induced H_2_ evolution system with supramolecular ZnP hexagonal nanocylinders that encapsulate Pt-colloid-deposited TiO_2_ nanoparticles in the internal cavity. Neither of these studies are concerned with the electrochemical water-splitting properties of Zn-metalloporphyrin. However, the third identified publication, by Seo et al. [38], describes an electrocatalytic system based on a porphyrin monolayer on atomically thin graphene. ZnP was part of the investigated series of metalloporphyrins and, in this case, the observed HER overpotential in a 0.5 M H_2_SO_4_ solution and at i = −3 mA/cm^2^ was ~0.56 V. No reported studies have been identified in which the HER and OER electrocatalytic properties of CoP were investigated. Nevertheless, the *para-*hydroxyl substituted Co(II) 5,10,15,20-tetrakis(4-hydroxyphenyl)-porphyrin structure was the subject of two studies relevant to the water-splitting domain. In one of them, Sun et al. [39] reported that Ni(II) and Co(II) 5,10,15,20-tetrakis(4-hydroxyphenyl)-porphyrin multilayers on reduced graphene oxide sheets behave as a bifunctional catalyst for the OER and the oxygen reduction reaction in an alkaline medium. Lastly, Wang et al. [40] obtained semiconductor photoanodes by decorating BiVO_4_ with graphene oxide and Co-porphyrins, including Co(II) 5,10,15,20-tetrakis(4-hydroxyphenyl)-porphyrin, and found that the photoelectrochemical water-splitting performance depends on the employed Co-porphyrin structure.

Unlike the mentioned studies, the current investigation evaluates the HER and OER electrocatalytic properties of graphite electrodes modified with one, two, and three layers of the specified metalloporphyrins, drop-casted from several organic solvents in electrolyte solutions covering a wide pH range. Furthermore, a morphological analysis focused on the self-assembled aggregates of the two species on a solid support was carried out as well, since the characteristics of these structures can significantly affect the catalytic activity of the electrodes.

## 2. Materials and Methods

### 2.1. Materials and Reagents

The syntheses and full characterisation of Zn(II) 5,10,15,20-tetrakis(4-pyridyl)-porphyrin (ZnP) and Co(II) 5,10,15,20-tetrakis(3-hydroxyphenyl)-porphyrin (CoP) have been previously reported [26,34], and their chemical structures are presented in Scheme S1 from the Supplementary Material File.

The reagents used in the electrochemical study of the two porphyrin derivatives were dimethylsulfoxide (DMSO), acetonitrile (CH_3_CN), benzonitrile (PhCN), potassium hydroxide, potassium chloride, and sulphuric acid 98% purchased from Merck (Darmstadt, Germany); N,N-dimethylformamide (DMF), dichloromethane (DCM), tetrahydrofuran (THF), and potassium hexacyanoferrate(III) provided by Sigma Aldrich (Saint Louis, MO, USA); ethanol (EtOH) from Honeywell (Charlotte, NC, USA); and acetone from Chimreactiv (Bucharest, Romania). Laboratory-obtained-double-distilled water was employed to prepare all aqueous solutions. The conductive graphite used to manufacture the electrodes was type SW.114 spectroscopic graphite from “Kablo Bratislava”, National Corporation “Electrocarbon Topolcany” Factory (Bratislava, Slovakia).

### 2.2. Manufacturing of the Working Electrodes

Polyethylene tubes were filled with graphite rods (ø = 6 mm) that were thermally treated (at 180 °C) until they became tightly attached to the tubes and only the rod ends remained bare. During the electrochemical experiments, one rod end was connected to the potentiostat, while the other was modified with each metalloporphyrin and immersed into the electrolyte solution. The modification procedure consisted of several stages. First, the surface of the graphite substrate was polished with silicon carbide paper with grit sizes of 800 and 1200 and felt. Second, the polished surface was washed with double-distilled water, ethanol, and acetone, and left to dry at 23 ± 2 °C. Third, the porphyrin solutions were prepared by dissolving the organic compounds in organic solvents with different polarities. According to Snyder et al. [41] and Szabadai et al. [42], the order of decreasing polarity for the employed solvents is DMSO > DMF > CH_3_CN > PhCN > EtOH > THF > DCM. The 0.15 mM porphyrin solutions were obtained following a 40 min ultrasonication treatment. Fourth, the polished graphite substrates were modified by drop-casting a 10 µL solution volume on their surfaces that was subsequently allowed to dry at 40 °C for 4 h and at 23 ± 2 °C for 20 h. This stage was required to deposit one metalloporphyrin layer on the graphite substrate and was repeated to obtain two and three such layers. The manufactured electrodes were labelled as presented in Table 1.

### 2.3. Electrochemical Experiments

The ensemble used to perform the electrochemical experiments consisted of a Voltalab PGZ 402 potentiostat from Radiometer Analytical (Lyon, France) and a glass cell equipped with three electrodes. The auxiliary electrode was a Pt plate (S_geom_ = 0.8 cm^2^) and the reference was the Ag/AgCl (sat. KCl) electrode. Each metalloporphyrin-modified graphite electrode—and an unmodified one (G_0_)—was utilized as the working electrode (S_geom_ = 0.28 cm^2^). The electrolyte solutions used to evaluate the OER and HER electrocatalytic properties of the manufactured electrodes were selected after testing the solubility of the two porphyrin species in a series of solutions—0.5 M H_2_SO_4_, 0.1 M H_2_SO_4_, 0.1 M KCl, 0.1 M KOH, and 1 M KOH—covering a wide pH range. It was found that ZnP was insoluble in the neutral and alkaline environments, while CoP was insoluble in neutral and acidic media. The experiments were performed in unstirred solutions, and prior to each HER investigation these were thoroughly deaerated with high-purity N_2_. Based on literature data [43], the iR-corrected polarisation curves recorded during the OER and HER studies were obtained at a scan rate (v) of 1 and 5 mV/s, respectively.

Except where otherwise stated, the current density (i) values relate to the geometric current density, while the electrochemical potential (E) values are represented vs. the reversible hydrogen electrode (RHE). The equations used to express the E values against the RHE and to calculate the values of other parameters relevant to the study, namely, the O_2_ and H_2_ evolution overpotentials (η_O2_ and η_H2_), the Tafel slope, and the electroactive surface area (EASA) [44,45,46], are shown in the Supplementary Material File as Equations (S1)–(S5).

### 2.4. Electron Microscopy Characterisation

An Inspect S scanning electron microscope (SEM) from FEI Company (Hillsboro, OR, USA) was used in high-vacuum mode to obtain SEM micrographs on the surface of the metalloporphyrin-modified graphite electrodes identified during the OER and HER experiments as having the best electrocatalytic properties. A Titan G2 80–200 transmission electron microscope from the same manufacturer was operated in TEM and scanning TEM (STEM) modes, at a 200 kV acceleration voltage, in order to analyse specimens prepared by drop-casting a 3 µL volume from each 0.15 mM metalloporphyrin solution employed in the electrochemical experiments on TEM copper grids covered with a continuous and amorphous carbon film.

## 3. Results and Discussions

### 3.1. TEM/STEM Characterisation of Metalloporphyrin Specimens

**Figure 1:**
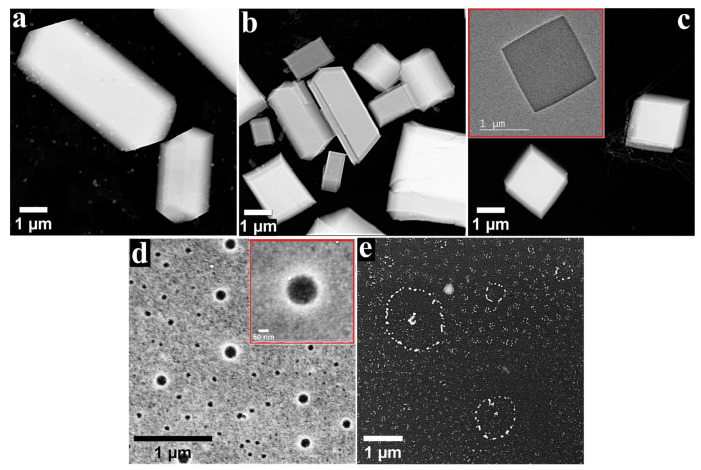
STEM images recorded for ZnP drop-casted on TEM grids from solutions obtained using the following solvents: (**a**) DMSO, (**b**) DMF, (**c**) PhCN, (**d**) THF, and (**e**) DCM. The inset in (**c**) shows the TEM image of a ZnP aggregate, and the inset in (**d**) presents a STEM image recorded at a higher magnification.

**Figure 2:**
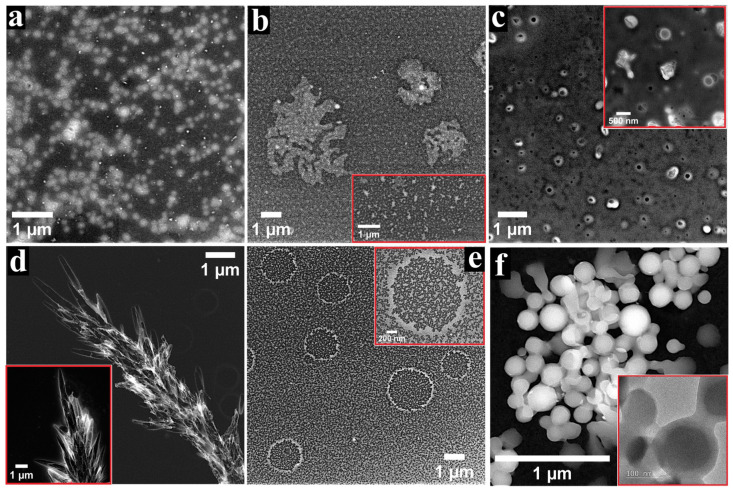
STEM images recorded for CoP drop-casted on TEM grids from solutions obtained using the following solvents: (**a**) DMSO, (**b**) DMF, (**c**) CH3CN, (**d**) PhCN, (**e**) EtOH, and (**f**) THF. The insets are STEM and TEM images that provide additional information about the specimens.

Through the process of molecular self-assembly, porphyrins in general and metalloporphyrins in particular have the ability to form well-defined aggregates that result from the non-covalent interactions between spontaneously associated molecules [30]. The self-assembly process can take place in solution or at interfaces, and the aggregates often have different properties from the molecules comprising them [47,48], including properties relevant to the water-splitting domain [49]. In order to outline the morphology of the structures developed from the self-assembly of the ZnP and CoP molecules, a TEM study was conducted on the specimens obtained by applying the metalloporphyrins on nonpolar carbon films from organic solvents with different polarity. The recorded images are presented in Figure 1 and Figure 2, and they reveal several types of architectures.

The image recorded on the ZnP specimen obtained using DMSO as a solvent (Figure 1a) shows irregular formations with micrometric lengths and widths, sprinkled with small particles sized between 30 and 180 nm. Irregular aggregates were also observed when the solvent was DMF (Figure 1b), but they were mixed with cuboid-like structures. While the length and width of most of the identified structures were micrometric, for some of the architectures resembling cuboids these dimensions were in the submicrometric range. Another similarity between this specimen and the previously discussed one consisted in the presence of small particles on the surface of the assemblies, while a difference was provided by the existence of crevices on the surface of some of the structures from the former sample. When applied from benzonitrile, the ZnP molecules organized as aggregates resembling cuboids and square plates (Figure 1c and inset). The measurements conducted for several cuboids revealed widths between 1.38 and 1.7 µm and thicknesses in the 0.2–0.44 µm range. It has been reported that complex porphyrin architectures comprise simpler ones, resulting from the side-by-side or face-to-face positioning of the molecules [50]. In the case of 3D aggregates—such as the ones observed for this specimen—these two types of assemblies, referred to as J-type and H-type arrangements, are both present [51].

In the case of the ZnP samples obtained using the solvents with the lowest polarity, the molecules no longer interacted to create irregular structures and cuboids but some very different aggregates instead (Figure 1d,e). Thus, when THF was employed as a solvent, a discontinuous metalloporphyrin layer covered the carbon film of the TEM grid. Ring-shaped formations revealed the substrate and appeared brighter than the surrounding ZnP deposits, probably due to a higher concentration of molecules. The ring diameters were in the 30–150 nm range. The inset in Figure 1d shows a magnified image of one of the rings (ø = 150 nm). When ZnP was applied from DCM, the observed aggregates were island-shaped and displayed a tendency to organize into circular arrangements (Figure 1e) due to their helical growth.

The TEM/STEM characterisation of the samples obtained using solutions of CoP dissolved in various organic solvents outlined a diversity of self-assembled architectures as well. Thus, when DMSO was employed as a solvent, the metalloporphyrin molecules formed cell-like 2D aggregates with submicrometric dimensions that co-existed with submicrometric particles (Figure 2a). The drop-casting of the Co-porphyrin from DMF (Figure 2b) resulted in discontinuous micrometric islands surrounded by the carbon film substrate patterned with porphyrin-based spots (inset in Figure 2b). When acetonitrile was used as a solvent, a discontinuous porphyrin layer covered the TEM grid and different types of arrangements were observed (Figure 2c), including irregular structures and cell-like formations (inset in Figure 2c)—all in the submicrometric domain. Figure 2d presents an image recorded for the specimen obtained by applying the Co-porphyrin species from benzonitrile. The molecules organized in patterns resembling flat crystalline bundles with micrometric lengths. The inset shows a fragment from such an arrangement. Micron-sized ring-like aggregates were obtained by drop-casting the CoP from ethanol (Figure 2e). For exemplification purposes, the inset in Figure 2e shows a magnified ring. Its thickness varied between 50 and 260 nm, being inconstant like the thickness of the other observed structures of the same type. The area between these architectures consisted in the carbon substrate covered to a high degree with irregularly shaped formations. Interestingly, the deposited material was noticed both outside and inside the rings, and this is important for identifying the de-wetting mechanism involved in their formation. According to the literature, there are two such mechanisms that explain the creation of ring-like assemblies, referred to as “coffee-stain” and “pinhole” [52,53]. The fact that a significant amount of material was located inside the rings is suggestive of the coffee-stain mechanism. As for the material between the rings, the pattern of the deposition is indicative of spinodal de-wetting [52]. When the Co-porphyrin was applied from THF, the identified aggregates were shaped as bundles of quasi-spherical structures (Figure 2f and inset). The performed measurements evidenced dimensions between 70 and 300 nm. Since the partially formed quasi-spheres observed during the investigation were not hollow, it is likely that the complete assemblies were also filled with metalloporphyrin molecules. These remarks are in agreement with other studies [54]. A general conclusion regarding the TEM/STEM analysis of the ZnP and CoP specimens is that a variety of aggregates were obtained by applying the two species on nonpolar carbon substrates from organic solvents with different polarity. Furthermore, a comparison among the samples that resulted by drop-casting ZnP and CoP from the same solvent revealed, in all cases, the presence of very different architectures. These are probably due to the interaction between the solvent, the substrate, and the elements specific to each metalloporphyrin (such as their substituents, their central metal ion, and their hydrophilic/hydrophobic balance).

The morphology of the aggregates is relevant for the electrochemical water-splitting field, since it has been reported that inhomogeneous electrode surfaces provide more catalytically active sites than homogeneous ones, and can thus lead to an improved charge transfer at the electrolyte/electrode interface during the OER and HER [55].

### 3.2. The OER and HER Electrocatalytic Activities of the ZnP- and CoP-Modified Electrodes

#### 3.2.1. OER and HER Investigations in 0.1 m Electrolyte Solutions

**Figure 3:**
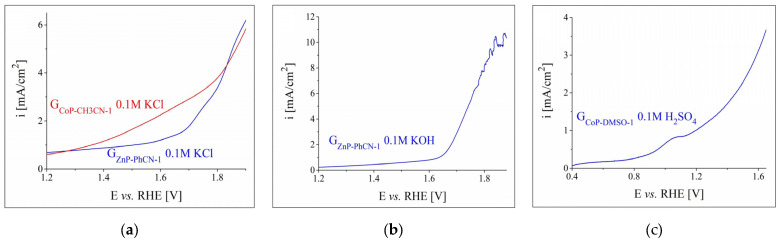
LSVs obtained during OER experiments on the most electrocatalytically active metalloporphyrin-modified electrodes, in: (**a**) 0.1 M KCl, (**b**) 0.1 M KOH, and (**c**) 0.1 M H_2_SO_4_ electrolyte solutions, at v = 1 mV/s.

Initially, linear sweep voltammograms (LSVs) were recorded in the anodic domain on the graphite electrodes modified with metalloporphyrins in order to study their OER electrocatalytic activity in aqueous electrolyte solutions with different pH values. The LSVs obtained in the 0.1 M KCl and 0.1 M KOH solutions on all the ZnP-modified electrodes are displayed in Figures S1 and S2 from the Supplementary Material File, while the ones traced in 0.1 M KCl and 0.1 M H_2_SO_4_ solutions on all the CoP-modified electrodes are shown in Figures S3 and S4. The curves recorded on the most electrocatalytically active metalloporphyrin-modified electrodes in the neutral, alkaline, and acidic media were selected from the four figures and are rendered in Figure 3. Figure 3a shows the overlapped voltammograms obtained on G_ZnP-PhCN-1_ and G_CoP-CH3CN-1_, which were identified as the most catalytically active electrodes in the neutral solution. G_ZnP-PhCN-1_ was also evidenced as having the highest catalytic activity for the OER in the alkaline medium (Figure 3b) and it attained the highest i value in the considered E range. The LSV traced on G_CoP-DMSO-1_ in an acidic environment can be seen in Figure 3c. This electrode was the most active in the specified medium and it reached the highest i value in the studied potential range. A comparison between the curves from Figure 3 reveals that the modified electrodes investigated in 0.1 M KCl solution displayed the lowest OER catalytic activity. Thus, no further experiments were performed concerning their electrocatalytic properties for the specified half-cell reaction in the neutral medium. The electrodes that exhibited the highest catalytic activity in alkaline and acidic environments were subsequently evaluated in more concentrated electrolyte solutions.

**Figure 4:**
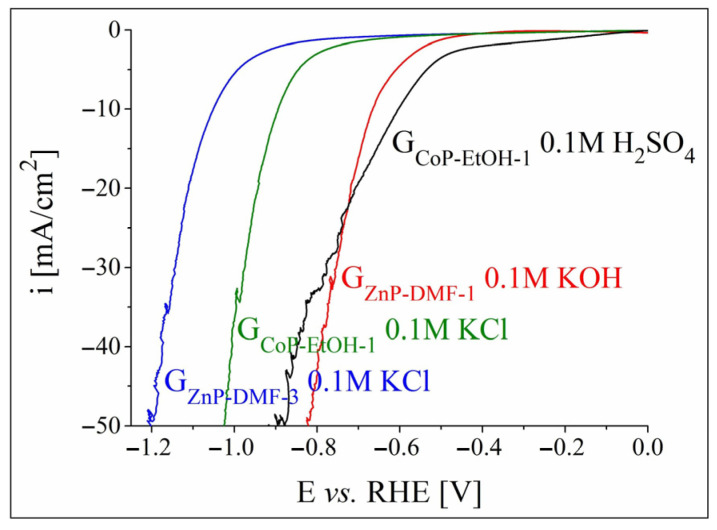
Cathodic polarisation curves recorded in 0.1 M electrolyte solutions for the most electrocatalytically active ZnP- and CoP-modified electrodes at v = 5 mV/s.

Polarisation curves were also recorded on the metalloporphyrin-modified graphite electrodes in the cathodic domain in order to study their HER electrocatalytic activity. The voltammograms traced in 0.1 M solutions are shown in Figures S5–S8 from the Supplementary Material File. Figures S5 and S6 present the experimental results obtained in neutral and alkaline solutions on all the ZnP-modified electrodes, while Figures S7 and S8 display the data acquired in neutral and acidic media on all the CoP-based electrodes. The highest catalytic activities for the HER were exhibited by G_ZnP-DMF-3_ and G_CoP-EtOH-1_ in 0.1 M KCl, G_ZnP-DMF-1_ in 0.1 M KOH, and G_CoP-EtOH-1_ in 0.1 M H_2_SO_4_. The LSVs for these electrodes are overlapped in Figure 4. The HER overpotential values were determined at i = –10 mA/cm^2^—a current density value at which η_H2_ is often specified [43,56,57]—as: 1.05 V for G_ZnP-DMF-3_ and 0.89 V for G_CoP-EtOH-1_ in the neutral medium, 0.67 V for G_ZnP-DMF-1_ in the alkaline medium, and 0.6 V for G_CoP-EtOH-1_ in the acidic electrolyte solution. Since the electrodes investigated in the neutral environment exhibited the highest η_H2_ values, they were not additionally studied in terms of their HER catalytic activity. The remaining two electrodes were subsequently tested in strong alkaline and strong acidic media.

As a general observation, the results of the OER and HER experiments carried out in the 0.1 M electrolyte solutions on all the ZnP- and CoP-based electrodes outline the fact that their electrocatalytic activity depended on the solutions drop-casted on the surface of the graphite substrate, obtained using solvents with different polarities. Furthermore, for most electrodes, the number of applied layers had a noticeable impact on their catalytic performance. 

Prior to continuing the evaluation of the OER and HER catalytic properties of the selected metalloporphyrin-modified electrodes, for which the voltammograms shown in Figure 3 and Figure 4 had been obtained, they were further electrochemically characterised in order to estimate their EASA and the diffusion coefficient of the hexacyanoferrate(III) ions. Thus, cyclic voltammograms were recorded at increasing scan rate values (v = 50, 100, 150, 200, 250, 300 and 350 mV/s) in a 1 M KNO_3_ electrolyte solution with and without 4 mM K_3_[Fe(CN)_6_]. The experimental data were used in the Randles–Sevcik equation [58,59], and the calculated values of the two parameters are displayed in Table 2. Each value is an average obtained from the experiments performed on two electrodes of the same type and is rendered with the standard deviation.

A high diffusion coefficient is a desirable electrochemical property for an electrode, since the higher the value, the faster the diffusion. The obtained diffusion coefficient values are higher than the tabulated ones published by Konopka et al. [60], determined using a thin-layer electrochemical cell with twin working Pt electrodes, but they are similar to the value estimated for a previously reported metalloporphyrin-modified electrode [44]. As can be seen in Table 2, the diffusion coefficient values for the porphyrin-based electrodes are higher than that of G_0_, which means that the self-assembled aggregates present on the substrate surface enhance diffusion. It should also be pointed out that in the case of G_ZnP-DMF-1_ and G_ZnP-DMF-3_, both the EASA and diffusion coefficient values are higher for the former, meaning that the drop-casting of more than one Zn(II) porphyrin layer from DMF leads to samples with worsened electrochemical properties. Therefore, the electrode-manufacturing procedure should involve the application of a single metalloporhyrin coat. Regarding the diffusion coefficient values calculated for the specimens obtained using one layer, they are within the same order of magnitude and the differences between them are not substantial, indicating that the properties of the self-assembled structures do not have a major impact on the parameter in question.

**Figure 5:**
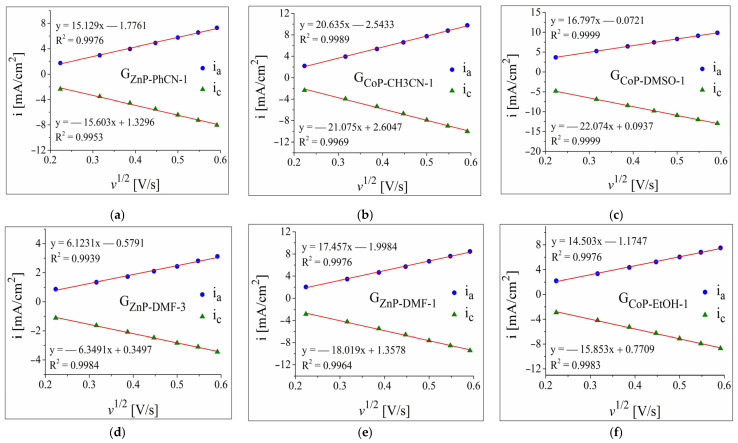
The plot of the anodic and cathodic peak current densities vs. the square root of the scan rate for the following electrodes: (**a**) G_ZnP-PhCN-1_, (**b**) G_CoP-CH3CN-1_, (**c**) G_CoP-DMSO-1_, (**d**) G_ZnP-DMF-3_, (**e**) G_ZnP-DMF-1_, and (**f**) G_CoP-EtOH-1_. The data were obtained from cyclic voltammograms recorded in 1 M KNO_3_ solution containing 4 mM K_3_[Fe(CN)_6_].

The cyclic voltammetry data resulting from studying the modified electrodes were further employed to represent the plots of the anodic and cathodic peak current densities vs. the square root of the scan rate (Figure 5). Without exception, the absolute values of the peak current densities increase with the scan rate, and this behaviour is typical of a diffusion-controlled electron transfer process [59].

#### 3.2.2. OER and HER Investigations in Strong Alkaline and Acidic Electrolyte Solutions

**Figure 6:**
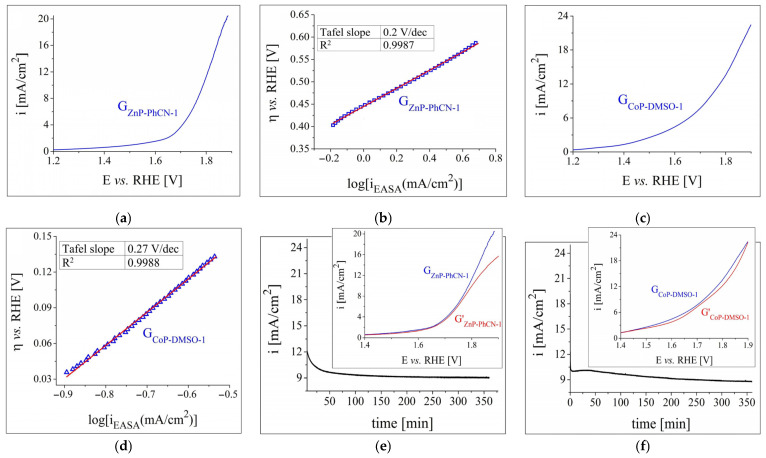
(**a**) Anodic polarisation curve recorded in 1 M KOH electrolyte solution on the G_ZnP-PhCN-1_ electrode at v = 1 mV/s. (**b**) The Tafel plot for the G_ZnP-PhCN-1_ sample in 1 M KOH solution. (**c**) Anodic polarisation curve traced in 0.5 M H_2_SO_4_ electrolyte solution on the G_CoP-DMSO-1_ electrode. (**d**) The Tafel plot for the G_CoP-DMSO-1_ sample in 0.5 M H_2_SO_4_ solution. (**e**) The i–time curve recorded on the G_ZnP-PhCN-1_ electrode in 1 M NaOH solution and inset with the LSVs obtained on the same specimen, before (G_ZnP-PhCN-1_) and after (G′_ZnP-PhCN-1_) the stability test. (**f**) The i–time curve recorded on the G_CoP-DMSO-1_ electrode in 0.5 M H_2_SO_4_ solution and inset with the voltammograms traced on the same specimen, before (G_CoP-DMSO-1_) and after (G′_CoP-DMSO-1_) the stability test.

The anodic polarisation curve obtained for G_ZnP-PhCN-1_ in 1 M KOH solution is presented in Figure 6a. A comparison with the LSV traced on the same electrode but in a lower pH electrolyte (Figure 3b) reveals that for all the recorded current density values the OER overpotential was smaller at the higher concentration. In addition, for the same potential range in which the voltammogram from Figure 3b was obtained, the increase in the concentration resulted in a higher maximum current density value. Such observations have been previously reported for other electrodes modified with water-splitting catalytic materials [46,61], including porphyrins [55]. The OER kinetics at the interface between the G_ZnP-PhCN-1_ electrode and the 1 M KOH electrolyte solution were also studied. In order to represent the Tafel plot (Figure 6b), the current density was normalised by the calculated EASA value of the investigated sample (i_EASA_). The Tafel equation [62] was used to determine a Tafel slope value of 0.2 V/dec.

Figure 6c shows the LSV obtained on the G_CoP-DMSO-1_ electrode in a strong acidic environment. The increase in the electrolyte solution concentration from 0.1 M (Figure 3c) to 0.5 M H_2_SO_4_ resulted in a smaller OER overpotential and higher current density values. η_O2_ was determined at i = 10 mA/cm^2^, in accordance with other studies [43,63], and was found to be 0.51 V. This value is smaller than the one calculated for the G_ZnP-PhCN-1_ specimen (of 0.56 V), studied in the 1 M KOH solution, and it indicates a higher OER electrocatalytic activity. However, the Tafel slope for G_CoP-DMSO-1_, obtained from the Tafel plot presented in Figure 6d, was higher than the one determined for G_ZnP-PhCN-1_, which points to the faster OER kinetics of the latter electrode [64].

Both G_ZnP-PhCN-1_ and G_CoP-DMSO-1_ were also evaluated in terms of their electrochemical stability. Thus, a 6 h chronoamperometric test was performed for each electrode at the potential value corresponding to i = 10 mA/cm^2^. The recorded current density–time curves are shown in Figure 6e,f, and they indicate the relative stability of the samples under the experimental conditions. However, in the case of the G_ZnP-PhCN-1_ electrode, the voltammogram traced after the stability test (inset in Figure 6e) increasingly differed in shape compared to the initial LSV as the current density increased, while it can be seen that the G′_CoP-DMSO-1_ curve, obtained using the G_CoP-DMSO-1_ electrode, does not deviate as much from the one recorded before the experiment (inset in Figure 6f).

**Figure 7:**
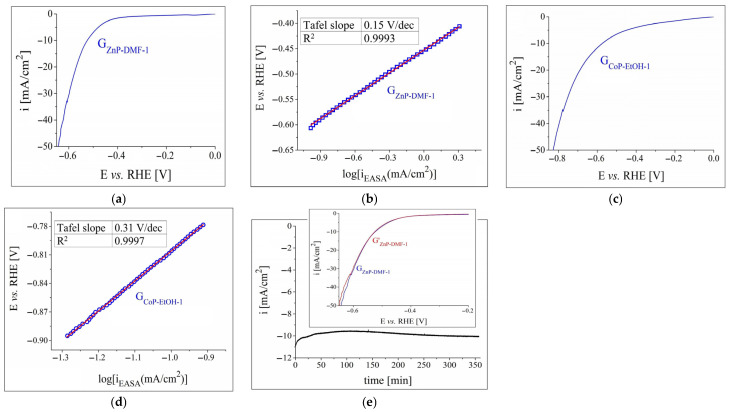
(**a**) Cathodic polarisation curve recorded in 1 M KOH electrolyte solution on the G_ZnP-DMF-1_ electrode at v = 5 mV/s. (**b**) The Tafel plot for the G_ZnP-DMF-1_ sample in 1 M KOH solution. (**c**) Cathodic polarisation curve obtained in 0.5 M H_2_SO_4_ electrolyte solution on the G_CoP-EtOH-1_ electrode. (**d**) The Tafel plot for the G_CoP-EtOH-1_ sample in 0.5 M H_2_SO_4_ solution. (**e**) The i–time curve recorded on the G_ZnP-DMF-1_ electrode in 1 M NaOH solution and inset with the LSVs traced on the same specimen, before (G_ZnP-DMF-1_) and after (G′_ZnP-DMF-1_) the stability test.

The results of the HER investigations performed on G_ZnP-DMF-1_ in a strong alkaline medium and on G_CoP-EtOH-1_ in a strong acidic medium are presented in Figure 7. The cathodic polarisation curves obtained on these two electrodes are shown in Figure 7a,c, while the Tafel plots determined for them are displayed in Figure 7b,d. The η_H2_ and Tafel slope values are rendered in Table 3, and allow for the following observations: (a) the more concentrated electrolyte solutions improved the HER catalytic activity of the samples, which is reflected in the smaller η_H2_ values; (b) the overpotential decrease was more significant for the ZnP-modified electrode than for the CoP-modified one, making it the most electrocatalytically active specimen with respect to the HER; and (c) the Tafel slope value for G_ZnP-DMF-1_ was notably smaller than the one determined for G_CoP-EtOH-1_, evidencing more favourable HER kinetics. Nevertheless, the slope is >120 mV/dec, which may indicate the occurrence of secondary processes, such as reduction reactions at the catalytic material’s surface or electrochemical H_2_ absorption [65].

Since the data outlined the Zn metalloporphyrin-based electrode as the most catalytically performant for the HER, the sample was further studied in terms of its electrochemical stability. As the i-time curve shows (Figure 7e), the −10 mA/cm^2^ current density was reached after 28 min. The i-value continued to increase to −9.5 mA/cm^2^, and it subsequently decreased back to −10 mA/cm^2^ (after 290 min). This value remained almost constant until the end of the experiment. The inset in Figure 7e presents the LSVs obtained before and after the chronoamperometric test, and it can be noticed that there is no significant difference between them until i = −33 mA/cm^2^. The changes of shape at the lower current densities can, at least in part, be attributed to the strong H_2_ evolution.

The study of the ZnP- and CoP-modified electrodes in terms of their OER and HER electrocatalytic activities revealed that G_CoP-DMSO-1_ and G_ZnP-DMF-1_ were, overall, more electrocatalytically performant than the other investigated modified samples. The surfaces of these electrodes were analysed by SEM before and after testing their electrochemical stability in order to examine the effect of the experimental conditions on the morphology of the porphyrin aggregates.

### 3.3. SEM Characterisation of the G_CoP-DMSO-1_ and G_ZnP-DMF-1_ Electrodes

**Figure 8:**
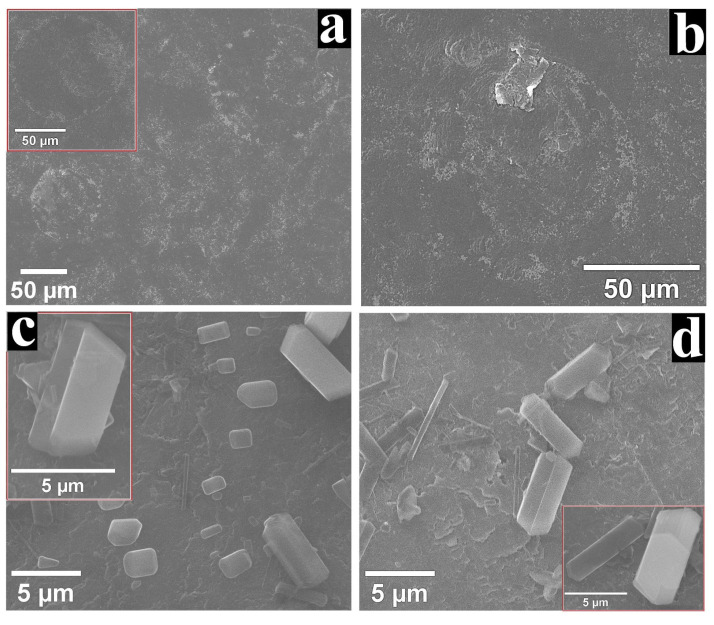
(**a**) SEM micrograph obtained on G_CoP-DMSO-1_ before the electrochemical stability experiment. Inset shows an aggregate identified on the specimen. (**b**) SEM image recorded on G_CoP-DMSO-1_ after the test. (**c**) SEM micrograph obtained on G_ZnP-DMF-1_ before the stability experiment. The inset presents an image at higher magnification. (**d**) SEM image recorded on G_ZnP-DMF-1_ after the test. The inset shows an image at higher magnification.

The SEM micrographs recorded on the surface of G_CoP-DMSO-1_ and G_ZnP-DMF-1_ are displayed in Figure 8. The images obtained on the surface of the Co-porphyrin-modified electrode before (Figure 8a and inset) and after (Figure 8b) the chronoamperometric study show aggregates with similar morphology. It can also be noted that the shape of these assemblies resembles the cell-like 2D structures revealed during the TEM/STEM study (Figure 2a) but are much bigger in size. In the case of the G_ZnP-DMF-1_ electrode, the structures present on its surface before the stability experiment (Figure 8c and inset) were similar to those following the test (Figure 8d and inset), as well as to the ones outlined during the TEM/STEM characterisation (Figure 1b). The results of the SEM analysis indicate that the metalloporphyrin aggregates that self-assembled on the graphite substrate’s surface did not undergo any significant morphological modifications in the course of the electrochemical stability test and this is a benefit for long-time usage.

### 3.4. Additional Observations Concerning the G_CoP-DMSO-1_ and G_ZnP-DMF-1_ Electrodes

The water-splitting electrocatalytic properties of metalloporphyrins have been the subject of several studies that have revealed the central metal cation as the catalytic centre [21]. For the two species that are the subject of the current study, the catalytic centres are the Zn^2+^ and Co^2+^ cations located in the Zn-N_4_ and Co-N_4_ sites of the porphyrin macrocycles. However, the catalytic performance of porphyrins is not provided by the metal ion alone, but by other factors as well. One of them is the previously specified ability of porphyrins to self-assemble into aggregates. In this respect, the SEM analysis of both the G_CoP-DMSO-1_ and G_ZnP-DMF-1_ samples revealed the unevenness of the depositions covering the graphite substrate. The disordered distribution of the ZnP structures (together with their edges and defects, such as the crevices observed on some of them) and the discontinuous CoP-based coating outline the inhomogeneous nature of the electrode surfaces, and inhomogeneities have been reported to increase the number of catalytically active sites exposed to the electrolyte solution [66,67]. Furthermore, the EASA value is directly proportional to the number of active sites involved in electrochemical reactions [68], and the calculated values for the two specimens are among the highest found in the study. 

Another property of metalloporphyrins relevant to their water-splitting catalytic performance is the electronegativity of the metal centre. The higher the electron affinity of the metal cation in the M-N_4_ site of the porphyrin macrocycle is, the stronger its electropositive doping effect on the carbon support becomes [38]. The electronegativities of both Zn and Co were higher than those of the C atoms comprising the graphite substrate, and this facilitated the charge transfer at the porphyrin–graphite interface. The OER and HER catalytic activity of metalloporphyrins is also affected by the electron-donating or electron-withdrawing properties of the functional moieties serving as their substituents [21]. ZnP has four electron-withdrawing pyridyl groups, while CoP has four electron-withdrawing hydroxyphenyl groups, due to their *meta* positioning. The HER experimental results revealed a ZnP-modified electrode as the most catalytically active, instead of a CoP-based one, despite the fact that Co is more electronegative than Zn and that the OH^–^ groups form intermolecular hydrogen bonds contribute to charge transport. Even in the case of the OER study, the differences between the G_CoP-DMSO-1_ and G_ZnP-PhCN-1_ samples were not very significant. The explanation for these observations likely includes the effects of the *meso*-substituents. The electron-withdrawing moieties are known to decrease the macrocycle’s electron density, leading to improved stability during catalysis and a positive shift in reduction potential. The outcome is a decreased energy cost for H_2_ evolution and an increased oxidising power in the case of OER catalysts [21,69].

The expected pH influence is in line with the obtained results. It is expected that the OER’s catalysis was more adequately achieved for the Co-porphyrin in a strong acidic medium because the four hydroxyl functional groups grafted on the porphyrin ring increase their tendency to form hydrogen bonds due to the substitution in the *meta* position. In a recent paper, it was noticed that the enhanced catalytic activity was due to the Co-porphyrin–OH intermediate [24].

Another possible explanation is the electronic effect of the substituents on the electronic structure at the cobalt centre [24]. Due to the already-known electron attractive property of OH groups in the *meta* position of the *meso*-phenyls [70] and to the surrounding hydrogen bonds, the electron densities on the Co ions decreased, and thus the nucleophilic attack of a water molecule was facilitated [19].

In the same manner, the highest HER catalytic activity can also be expected in an alkaline solution for the electrode covered with Zn-porphyrin because the four pyridyl groups grafted on the porphine ring are stable in this medium, but generate pyridinium N^+^H in an acidic environment, thus promoting dimerization and an undesired uniform and compact self-aggregation due to London’s dispersion force being the main binding force [71]. In addition, the positional disorder of the pyrrole NH atoms was demonstrated in pyridyl-substituted porphyrins [72] in normal and basic pH. Instead, the distorted porphyrin molecules obtained in acidic medium by the protonation of the N-pyridyl groups were packed more efficiently in adjacent stacks because of the weak C–H…N hydrogen bonds formed between β-pyrrole moieties and the pyridyl groups of the molecules. This type of compact arrangement diminishes the number of catalytically active sites in acidic media.

In order to compare the OER and HER electrocatalytic activity of the G_CoP-DMSO-1_ and G_ZnP-DMF-1_ specimens with other reported electrodes manufactured using Zn- and Co-porphyrins, a literature survey was conducted and presented in Table S1 from the Supplementary Material File that outlined a disproportionate number of studies performed on cobalt metalloporphyrins, as opposed to zinc-metalated ones, which have been more the subject of photocatalytic water-splitting investigations [49,73]. The findings indicate that the η_H2_ value for G_ZnP-DMF-1_ is either similar to or smaller than the values reported for electrodes modified only with Zn- and Co-porphyrins, while also being similar to several of the ones published for more complex Co-porphyrin-based catalysts. The same situation is observed when contrasting the η_O2_ value for G_CoP-DMSO-1_ with the ones exhibited by other electrodes manufactured using Co-porphyrins. The Tafel slopes determined for the two samples are among the highest identified, which suggests that in future water-splitting studies of these metalloporphyrins they should be utilized in combination with other materials (nickel phosphite, carbon black, and perovskites) so as to obtain a synergistic effect.

## 4. Conclusions

Two symmetrically substituted metalloporphyrins were investigated as heterogeneous catalysts for the two half-cell reactions involved in the water-splitting process in aqueous electrolyte solutions with different pH values. The porphyrin solutions obtained using organic solvents with different polarities were drop-casted on TEM grids and on graphite supports. The TEM/STEM characterisation revealed several types of self-assembled porphyrin aggregates, resulting from the interplay between the solvent’s polarity, the nonpolar carbon film substrate, and the properties of the differently substituted Zn- and Co-porphyrins. The study of the OER and HER electrocatalytic properties of the manufactured metalloporphyrin-based graphite samples revealed the most performant electrodes for each environment in which they were evaluated. The best overall results concerning OER catalysis were observed in a strong acidic medium for the sample manufactured by applying one layer of Co-porphyrin from DMSO, while the highest HER catalytic activity was evidenced in a strong alkaline solution on the electrode made with one layer of Zn-porphyrin drop-casted from DMF. In the first case, a η_O2_ value of 0.51 V (at i = 10 mA/cm^2^) and a Tafel slope value of 0.27 V/dec were determined, and in the second case, a η_H2_ value of 0.52 V (at i = −10 mA/cm^2^) and a Tafel slope value of 0.15 V/dec were obtained. The stability of the two electrodes was verified electrochemically and by an SEM analysis.

## Figures and Tables

**Table 1:** The labelling of the modified electrodes.

Electrode Label	Metallo- Porphyrin	Solvent	Applied Layers	Electrode Label	Metallo- Porphyrin	Solvent	Applied Layers
G_ZnP-DMSO-1_	ZnP	DMSO	1	G_CoP-DMSO-1_	CoP	DMSO	1
G_ZnP-DMSO-2_			2	G_CoP-DMSO-2_			2
G_ZnP-DMSO-3_			3	G_CoP-DMSO-3_			3
G_ZnP-DMF-1_		DMF	1	G_CoP-DMF-1_		DMF	1
G_ZnP-DMF-2_			2	G_CoP-DMF-2_			2
G_ZnP-DMF-3_			3	G_CoP-DMF-3_			3
G_ZnP-PhCN-1_		PhCN	1	G_CoP-CH3CN-1_		CH_3_CN	1
G_ZnP-PhCN-2_			2	G_CoP-CH3CN-2_			2
G_ZnP-PhCN-3_			3	G_CoP-CH3CN-3_			3
G_ZnP-THF-1_	ZnP	THF	1	G_CoP-PhCN-1_	CoP	PhCN	1
G_ZnP-THF-2_			2	G_CoP-PhCN-2_			2
G_ZnP-THF-3_			3	G_CoP-PhCN-3_			3
G_ZnP-DCM-1_		DCM	1	G_CoP-EtOH-1_		EtOH	1
G_ZnP-DCM-2_			2	G_CoP-EtOH-2_			2
G_ZnP-DCM-3_			3	G_CoP-EtOH-3_			3
				G_CoP-THF-1_		THF	1
				G_CoP-THF-2_			2
				G_CoP-THF-3_			3

**Table 2:** The calculated EASA and diffusion coefficient values.

Electrode Label	EASA [cm^2^]	Diffusion Coefficient [cm^2^/s]
G_0_	0.325 ± 0.007	9.35 × 10^–6^ ± 0.14 × 10^–6^
G_ZnP-PhCN-1_	0.8 ± 0.06	6.15 × 10^–5^ ± 0.24 × 10^–5^
G_ZnP-DMF-3_	0.422 ± 0.0014	1.525 × 10^–5^ ± 0.007 × 10^–5^
G_ZnP-DMF-1_	0.942 ± 0.015	7.7 × 10^–5^ ± 0.24 × 10^–5^
G_CoP-CH3CN-1_	0.948 ± 0.07	8.375 × 10^–5^ ± 0.62 × 10^–5^
G_CoP-EtOH-1_	1.19 ± 0.08	5.7 × 10^–5^ ± 0.35 × 10^–5^
G_CoP-DMSO-1_	0.94 ± 0.04	7.62 × 10^–5^ ± 0.72 × 10^–5^

**Table 3:** The η_H2_ and Tafel slope values for the G_ZnP-DMF-1_ and G_CoP-EtOH-1_ electrodes.

Electrode Label	η_H2_ at i = −10 mA/cm^2^	Tafel Slope
G_ZnP-DMF-1_	0.67 V, in 0.1 M KOH	0.15 V/dec, in 1 M KOH
	0.52 V, in 1 M KOH	
G_CoP-EtOH-1_	0.6 V, in 0.1 M H_2_SO_4_	0.31 V/dec, in 0.5 M H_2_SO_4_
	0.57 V, in 0.5 M H_2_SO_4_	

## Data Availability

The data presented in this study are available on request from the corresponding author.

## Supplementary Materials

The following supporting information can be downloaded at: https://www.mdpi.com/article/10.3390/nano12213788/s1, Scheme S1: The chemical structures of (a) Zn(II) 5,10,15,20-tetrakis(4-pyridyl)-porphyrin and (b) Co(II) 5,10,15,20-tetrakis(3-hydroxyphenyl)-porphyrin; Figure S1: Anodic polarisation curves recorded in 0.1 M KCl solution on the graphite electrodes modified with ZnP, applied from (a) DMSO, (b) DMF, (c) PhCN, (d) THF, and (e) DCM. The samples are labelled according to Table 1 from the main text. G0 is the unmodified graphite electrode; Figure S2: Anodic polarisation curves recorded in 0.1 M KOH solution on the graphite electrodes modified with ZnP, applied from: (a) DMSO, (b) DMF, (c) PhCN, (d) THF, and (e) DCM. The samples are labelled according to Table 1 from the main text. G0 is the unmodified graphite electrode; Figure S3: Anodic polarisation curves recorded in 0.1 M KCl solution on the graphite electrodes modified with CoP, drop-casted from: (a) DMSO, (b) DMF, (c) CH3CN, (d) PhCN, (e) EtOH, and (f) THF. The samples are labelled according to Table 1 from the main text. G0 is the unmodified graphite electrode; Figure S4: Anodic polarisation curves recorded in 0.1 M H2SO4 solution on the graphite electrodes modified with CoP, drop-casted from: (a) DMSO, (b) DMF, (c) CH3CN, (d) PhCN, (e) EtOH, and (f) THF. The samples are labelled according to Table 1 from the main text. G0 is the unmodified graphite electrode; Figure S5: Cathodic polarisation curves obtained in 0.1 M KCl solution on the graphite electrodes modified with ZnP, applied from: (a) DMSO, (b) DMF, (c) PhCN, (d) THF and (e) DCM. The samples are labelled according to Table 1 from the main text. G0 is the unmodified graphite electrode; Figure S6: Cathodic polarisation curves obtained in 0.1 M KOH solution on the graphite electrodes modified with ZnP, applied from: (a) DMSO, (b) DMF, (c) PhCN, (d) THF and (e) DCM. The samples are labelled according to Table 1 from the main text. G0 is the unmodified graphite electrode; Figure S7: Cathodic polarisation curves obtained in 0.1 M KCl solution on the graphite electrodes modified with CoP, drop-casted from: (a) DMSO, (b) DMF, (c) CH3CN, (d) PhCN, (e) EtOH, and (f) THF. The samples are labelled according to Table 1 from the main text. G0 is the unmodified graphite electrode; Figure S8: Cathodic polarisation curves obtained in 0.1 M H2SO4 solution on the graphite electrodes modified with CoP, drop-casted from: (a) DMSO, (b) DMF, (c) CH3CN, (d) PhCN, (e) EtOH, and (f) THF. The samples are labelled according to Table 1 from the main text. G0 is the unmodified graphite electrode; Table S1: The OER and HER activities of G_CoP-DMSO-1_, of G_ZnP-DMF-1_ and of several reported electrodes. References [38,39,44,45,46,65,74,75,76,77,78,79,80,81,82,83,84,85,86,87,88,89] are cited in the Supplementary Material File.
