# Supplementary material for: The pH Influence on the Water-Splitting Electrocatalytic Activity of Graphite Electrodes Modified with Symmetrically Substituted Metalloporphyrins
Source: Nanomaterials (Basel). 2022 Oct 27;12(21):3788. doi: 10.3390/nano12213788 (PMC9656975; doi:10.3390/nano12213788)
Supplement: Supplementary file 1 [file nanomaterials-12-03788-s001.zip › nanomaterials-1982884-supplementary.pdf]

nanomaterials

Supplementary material

The pH Influence on the Water-Splitting Electrocatalytic Activity of Graphite Electrodes Modified with Symmetrically Substituted Metalloporphyrins

Bogdan-Ovidiu Taranu [1,*] and Eugenia Fagadar-Cosma [2,*]

1 National Institute for Research and Development in Electrochemistry and Condensed Matter, Dr. A. Paunescu Podeanu Street No. 144, 300569 Timisoara, Romania
2 Institute of Chemistry "Coriolan Dragulescu", Mihai Viteazu Ave. 24, 300223 Timisoara, Romania
* Correspondence: b.taranu84@gmail.com (B.-O.T.); efagadar@yahoo.com or efagadarcosma@acad-icht.tm.edu.ro (E.F.-C.)

Scheme

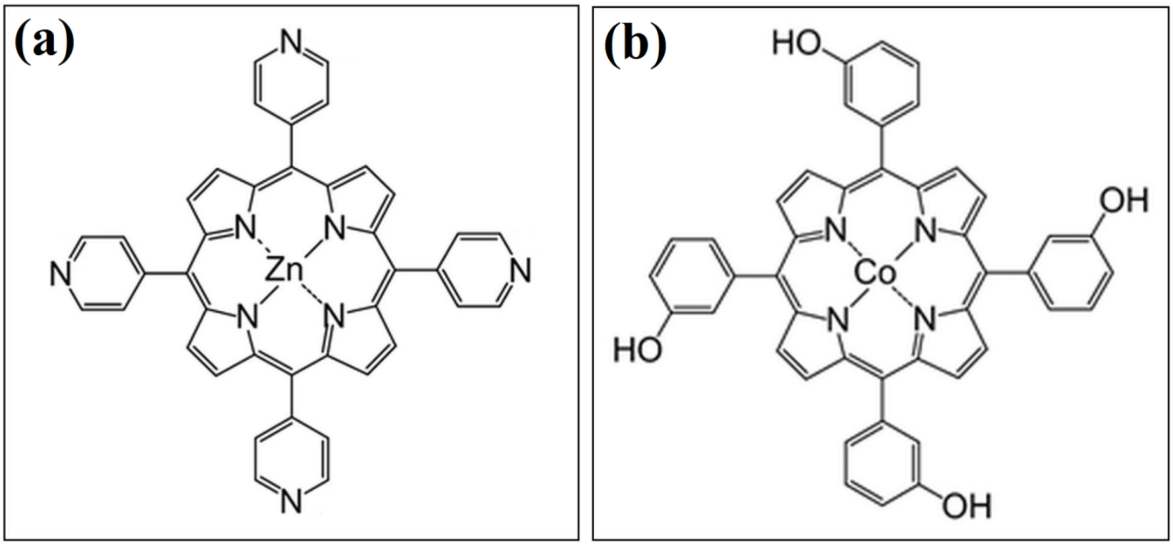

Scheme S1. The chemical structures of: (a) Zn(II) 5,10,15,20-tetrakis(4-pyridyl)-porphyrin and (b) Co(II) 5,10,15,20-tetrakis(3-hydroxyphenyl)-porphyrin.

Equations

Equations (S1) – (S5) [1-3] were employed in the present study as follows: Equation (S1) was used to express the electrochemical potential values measured *vs.* the Ag/AgCl(sat. KCl) reference electrode against the Reversible Hydrogen Electrode (RHE); the O_2 and H_2 overpotential values were calculated with Equations (S2) and (S3), and the Tafel slope was determined with Equation (S4).

$$E_{RHE} = E_{Ag/AgCl(sat.\ KCl)} + 0.059 \times pH + 0.197 \quad (S1)$$

$$\eta_{O2} = E_{RHE} - 1.23 \quad (S2)$$

$$\eta_{H2} = |E_{RHE}| \quad (S3)$$

$$\eta = b \times \log(i) + a \quad (S4)$$

Where: E_{RHE} is the reversible hydrogen electrode potential [V], $E_{Ag/AgCl(sat.\ KCl)}$ is the potential *vs.* the Ag/AgCl (sat. KCl) reference electrode [V], η_{O2} is the oxygen evolution overpotential and η_{H2} is the hydrogen evolution overpotential [V], η is either the oxygen

or the hydrogen evolution overpotential [V], i is the current density [mA/cm²] and b is the Tafel slope.

Equation (S5) is the Randles-Sevcik equation that can be employed to calculate the electroactive surface area (EASA) of an electrode, as well as the diffusion coefficient of the electroactive species.

$$I_p = (2.69 \times 10^5) \times n^{3/2} \times A \times D^{1/2} \times C \times v^{1/2} \quad \text{(S5)}$$

Where: I_p = the peak current [A]; n = the number of electrons involved in the redox process at T = 298 K; A = the surface area of the working electrode [cm²]; D = the diffusion coefficient of the electroactive species [cm²/s]; C = the bulk concentration of the electroactive species [M] and v = the scan rate [V/s].

For the ferrocyanide/ferricyanide redox system used in the present study, n = 1 and the theoretical value of the diffusion coefficient reported in the literature is 6.7 x 10^{-6} cm²/s [4,5].

Figures

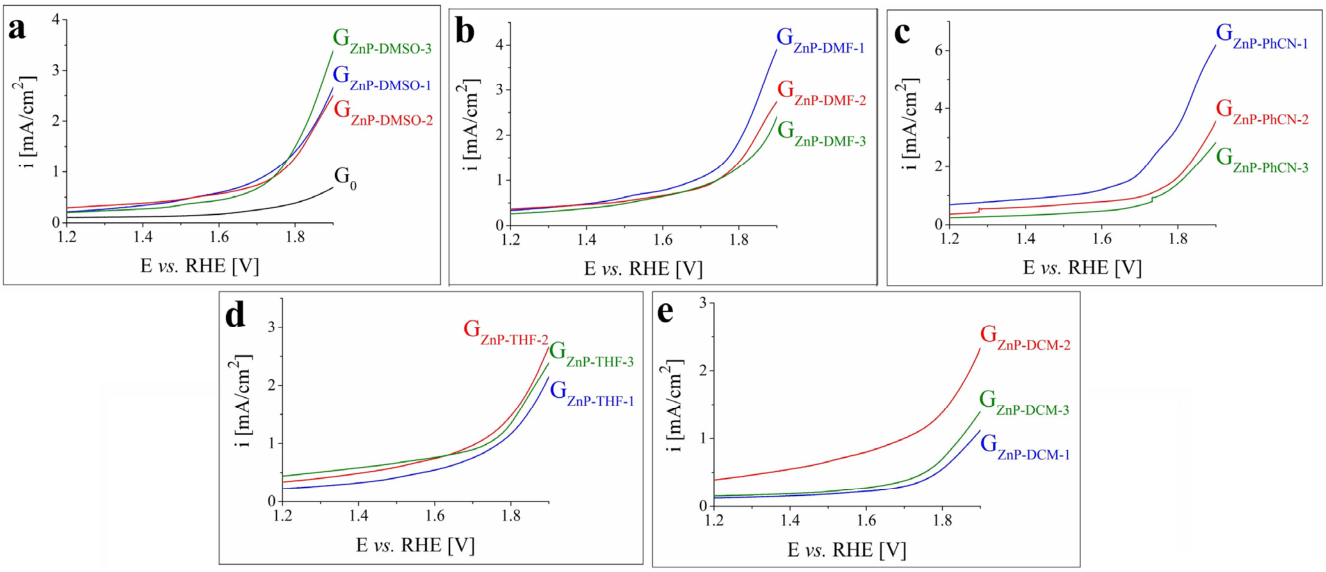

Figure S1. Anodic polarization curves recorded in 0.1 M KCl solution on the graphite electrodes modified with ZnP, applied from: (a) DMSO, (b) DMF, (c) PhCN, (d) THF and (e) DCM. The samples are labelled according to Table 1 from the main text. G_0 is the unmodified graphite electrode.

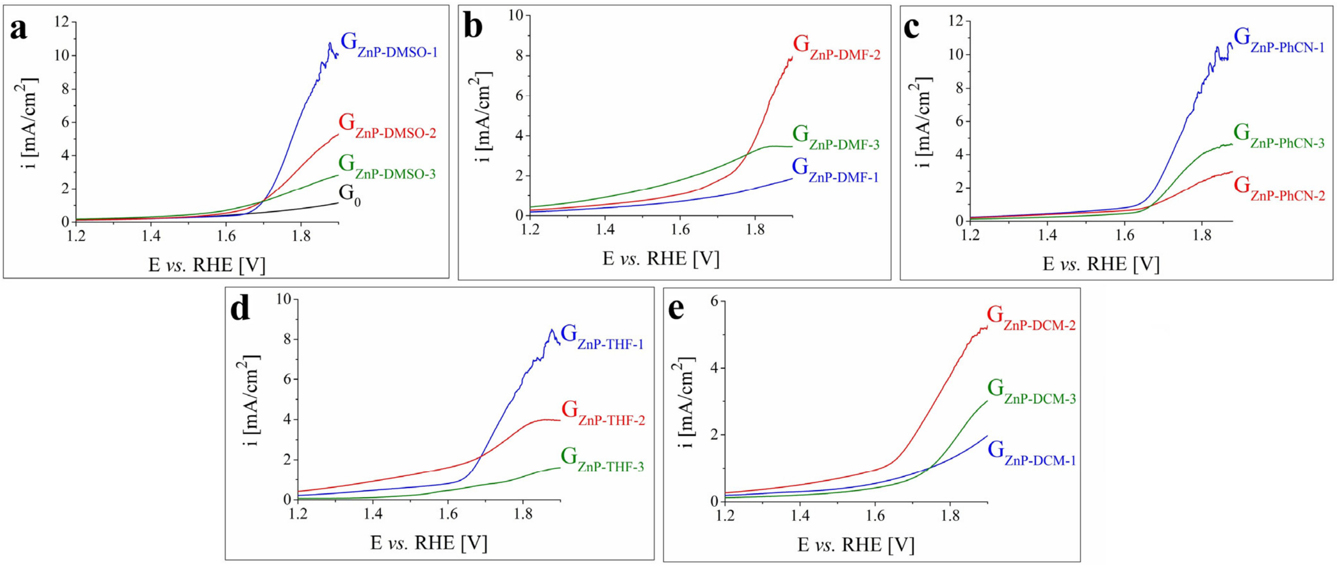

Figure S2. Anodic polarization curves recorded in 0.1 M KOH solution on the graphite electrodes modified with ZnP, applied from: (a) DMSO, (b) DMF, (c) PhCN, (d) THF and (e) DCM. The samples are labelled according to Table 1 from the main text. G_0 is the unmodified graphite electrode.

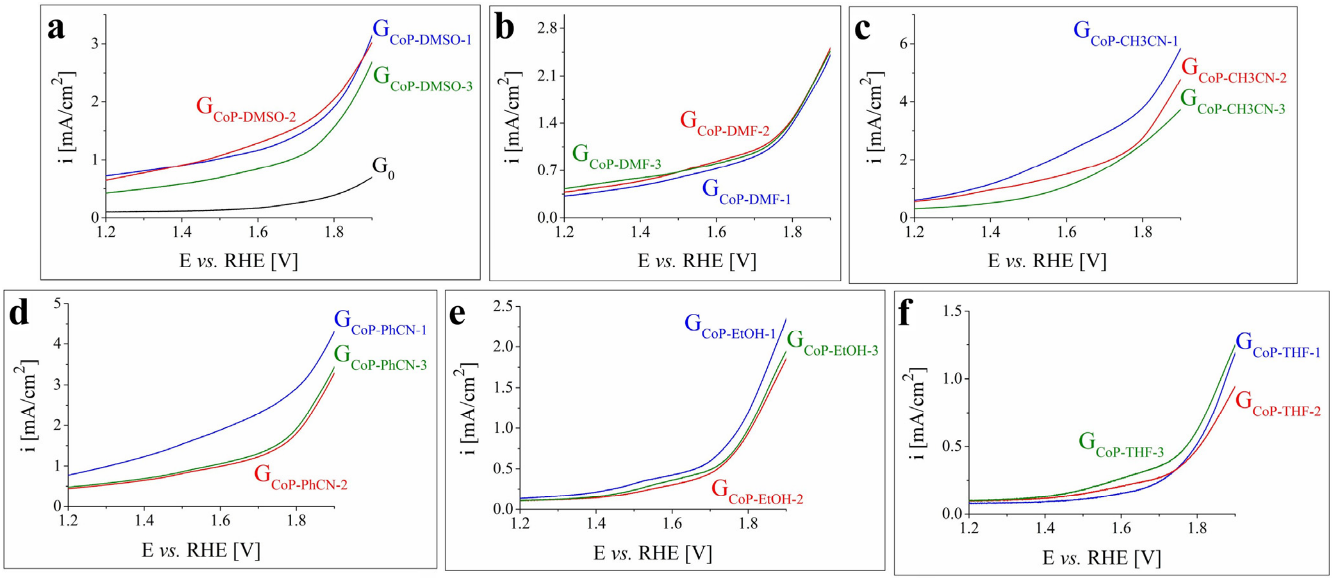

Figure S3. Anodic polarization curves recorded in 0.1 M KCl solution on the graphite electrodes modified with CoP, drop-casted from: (a) DMSO, (b) DMF, (c) CH_3CN, (d) PhCN, (e) EtOH and (f) THF. The samples are labelled according to Table 1 from the main text. G_0 is the unmodified graphite electrode.

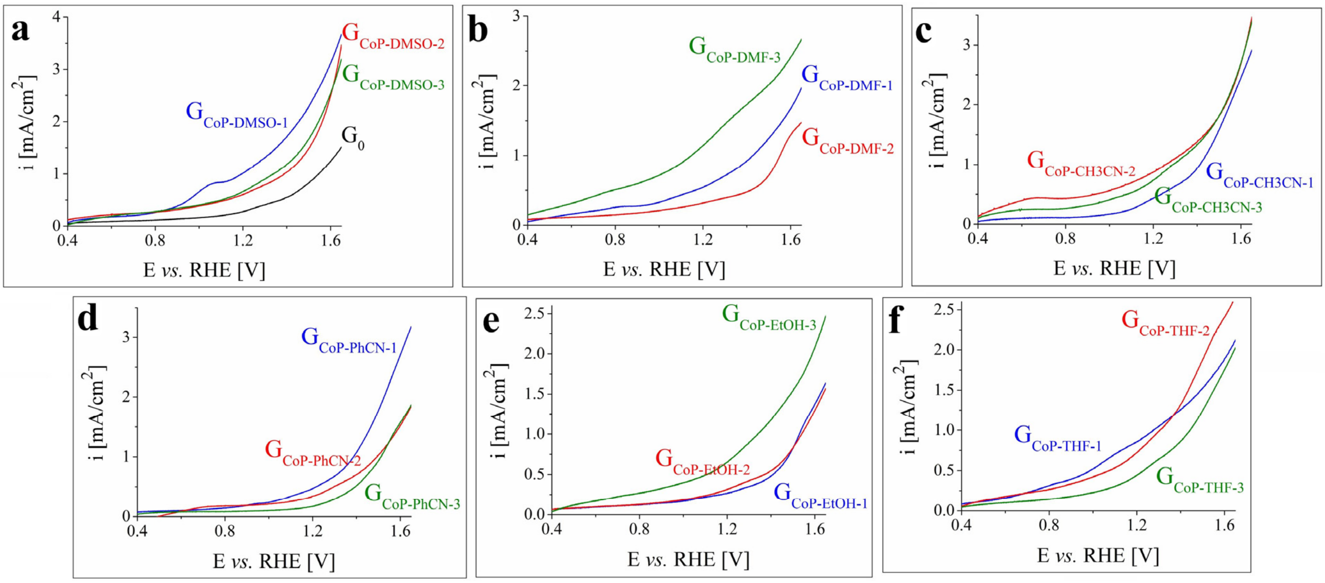

Figure S4. Anodic polarization curves recorded in 0.1 M H_2SO_4 solution on the graphite electrodes modified with CoP, drop-casted from: (a) DMSO, (b) DMF, (c) CH_3CN, (d) PhCN, (e) EtOH and (f) THF. The samples are labelled according to Table 1 from the main text. G_0 is the unmodified graphite electrode.

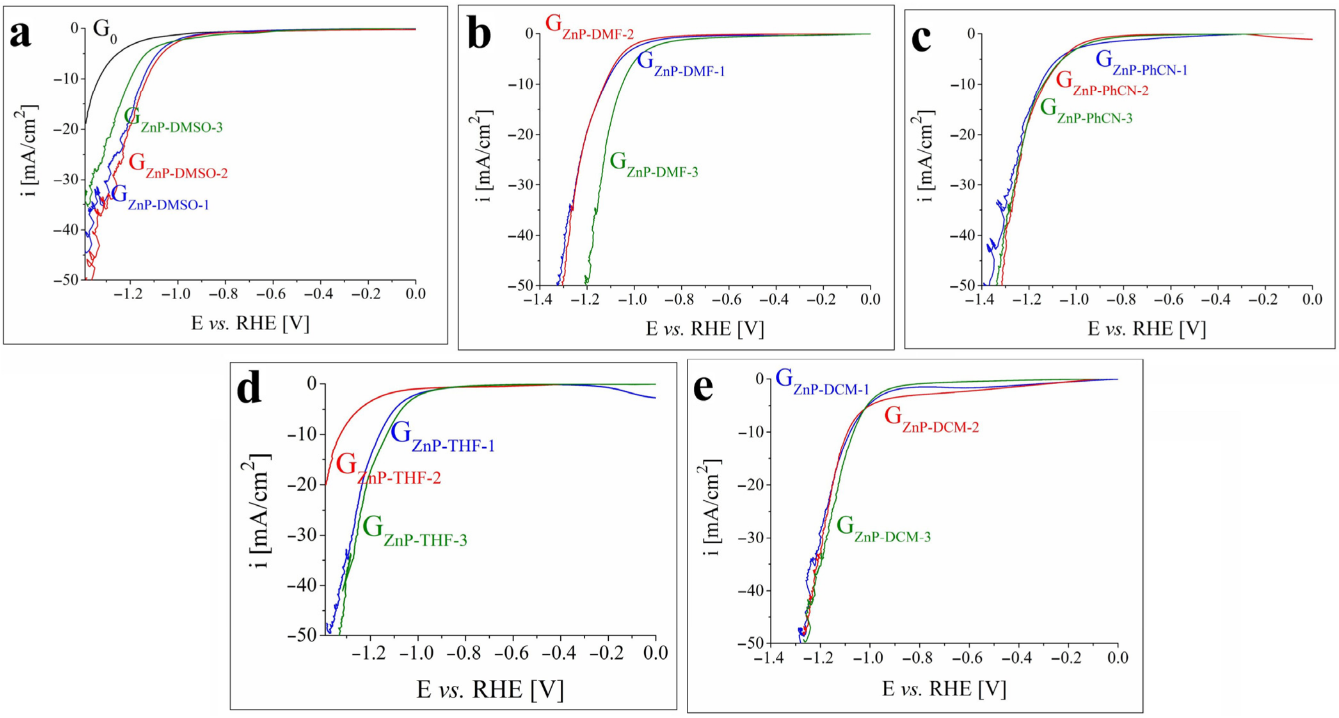

Figure S5. Cathodic polarization curves obtained in 0.1 M KCl solution on the graphite electrodes modified with ZnP, applied from: (a) DMSO, (b) DMF, (c) PhCN, (d) THF and (e) DCM. The samples are labelled according to Table 1 from the main text. G_0 is the unmodified graphite electrode.

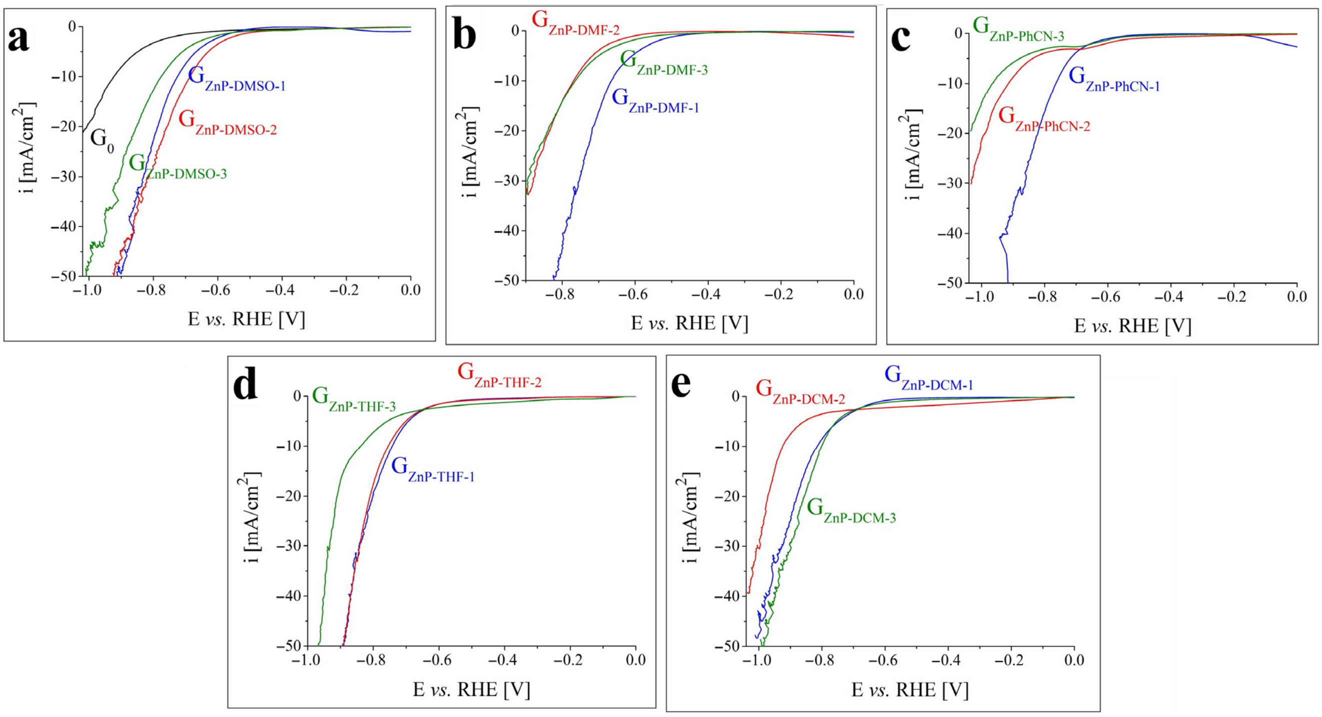

Figure S6. Cathodic polarization curves obtained in 0.1 M KOH solution on the graphite electrodes modified with ZnP, applied from: (a) DMSO, (b) DMF, (c) PhCN, (d) THF and (e) DCM. The samples are labelled according to Table 1 from the main text. G_0 is the unmodified graphite electrode.

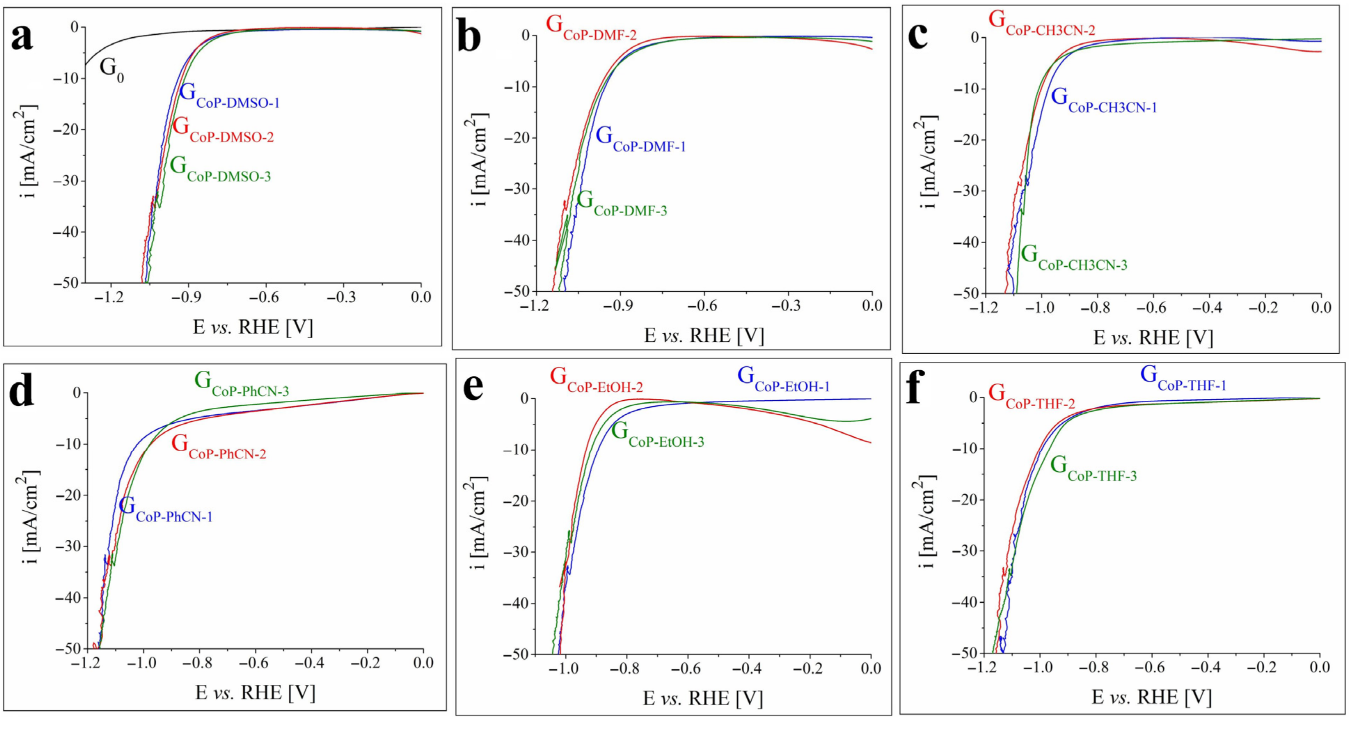

Figure S7. Cathodic polarization curves obtained in 0.1 M KCl solution on the graphite electrodes modified with CoP, drop-casted from: (a) DMSO, (b) DMF, (c) CH_3CN, (d) PhCN, (e) EtOH and (f) THF. The samples are labelled according to Table 1 from the main text. G_0 is the unmodified graphite electrode.

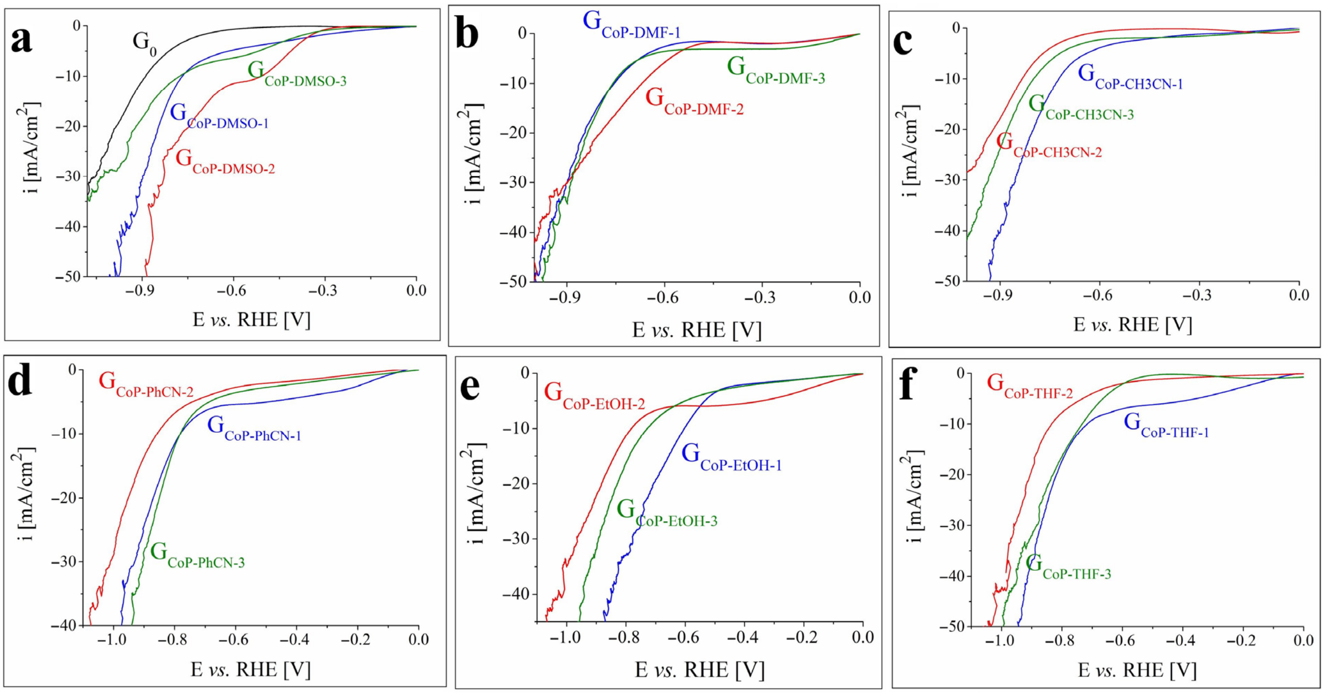

Figure S8. Cathodic polarization curves obtained in 0.1 M H_2SO_4 solution on the graphite electrodes modified with CoP, drop-casted from: (a) DMSO, (b) DMF, (c) CH_3CN, (d) PhCN, (e) EtOH and (f) THF. The samples are labelled according to Table 1 from the main text. G_0 is the unmodified graphite electrode.

Table

Table S1. The OER and HER activities of $G_{CoP\text{-}DMSO\text{-}1}$, of $G_{ZnP\text{-}DMF\text{-}1}$ and of several reported electrodes.

Catalyst	Substrate	Electrolyte	Overpotential [V]	Tafel slope [V/dec]	Catalysed reaction	Ref.
Zn-TPP [a]	graphene	0.5 M H_2SO_4	~ 0.48 at –3 mA/cm^2	-	HER	[6]
Zn-TAPP [b]	graphene	0.5 M H_2SO_4	~ 0.48 at –3 mA/cm^2	-	HER	[6]
Zn-TPyP [c]	graphene	0.5 M H_2SO_4	~ 0.56 at –3 mA/cm^2	-	HER	[6]
ZnTAPP-NA [d]	GCE [e]	1 M KOH	0.546 at –10 mA/cm^2	0.121	HER	[7]
ZnTAPP-NA	GCE	1 M KOH	-	0.313	OER	[7]
CoTAPP-NA [f]	GCE	1 M KOH	0.47 at –10 mA/cm^2	0.11	HER	[7]
CoTAPP-NA	GCE	1 M KOH	0.416 at 10 mA/cm^2	0.068	OER	[7]
CoTcPP/ZrP [g]	GCRDE [h]	0.1 M KOH	0.467 at 10 mA/cm^2	0.076	OER	[8]
$(Co–P)_{0.5}(Fe–P)_{0.5}$@CNT [i]	GCRDE	0.1 M KOH	0.42 at 10 mA/cm^2	-	OER	[9]
3,4,5-OMe-CoP/CNT [j]	GCE	1 M KOH	0.482 at 10 mA/cm^2	0.081	OER	[10]
2,4,6-OMe-CoP/CNT [k]	GCE	1 M KOH	0.5 at 10 mA/cm^2	0.09	OER	[10]
1/CNTs [l]	RDE [m]	1 M KOH	0.407 at 10 mA/cm^2	0.06	OER	[11]
2/CNTs [n]	RDE	1 M KOH	0.48 at 10 mA/cm^2	0.072	OER	[11]
1/MWCNT [o]	GCE	0.1 M PBS	0.79	-	OER	[12]

			at 10 mA/cm^2			
1/MWCNT	GCE	1 M KOH	0.48 at 10 mA/cm^2	-	OER	[12]
1/MWCNT/Py-Py [p]	GCE	0.1 M PBS	0.65 at 10 mA/cm^2	-	OER	[12]
1/MWCNT/Py-Py	GCE	1 M KOH	0.44 at 10 mA/cm^2	-	OER	[12]
CoTPP-SD [q]	CFP [r]	1 M KOH	0.475 at –10 mA/cm^2	-	HER	[13]
CoTPP-SD	CFP	1 M KOH	0.67 at 10 mA/cm^2	-	OER	[13]
CoCOP [s]	CFP	1 M KOH	0.31 at –10 mA/cm^2	0.161	HER	[13]
CoCOP	CFP	1 M KOH	0.35 at 10 mA/cm^2	0.151	OER	[13]
CoTCPP [t]	FTO/Ag	0.5 M H_2SO_4	0.666 at –10 mA/cm^2	0.264	HER	[14]
CoTCPP polymer [u]	FTO/Ag	0.5 M H_2SO_4	0.475 at –10 mA/cm^2	0.197	HER	[14]
PIZA-1-400 [v]	FTO	1 M KOH	0.43 at 10 mA/cm^2	0.052	OER	[15]
CeO_2@PIZA-1-400 [w]	FTO	1 M KOH	0.37 at 10 mA/cm^2	0.048	OER	[15]
CoTMPyP/ERGO [x]	GCE	0.1 M KOH	0.347 at –1 mA/cm^2	0.099	HER	[16]
CoTMPyP/ERGO	GCE	1 M KOH	0.315 at –1 mA/cm^2	0.096	HER	[16]
Co-MPPy-1 [y]	RDE	1 M NaOH	0.42 at 10 mA/cm^2	0.058	OER	[17]
Co-2DP [z]	Ti foil	1 M KOH	0.367 at –1 mA/cm^2	0.126	HER	[18]
rGO/(Ni^{2+}–THPP/Co^{2+}–THPP)$_8$ [aa]	RDE	1 M KOH	0.33 at 10 mA/cm^2	0.05	OER	[19]
CoP-2ph-CMP-800 [ab]	GCE	1 M KOH	0.36 at –10 mA/cm^2	0.121	HER	[20]
CoP-3ph-CMP-800 [ac]	GCE	1 M KOH	0.38 at –10 mA/cm^2	-	HER	[20]
CoP-4ph-CMP-800 [ad]	GCE	1 M KOH	0.44 at –10 mA/cm^2	-	HER	[20]
CoP-2ph-CMP-800	GCE	1 M KOH	0.37 at 10 mA/cm^2	0.086	OER	[20]
CoP-3ph-CMP-800	GCE	1 M KOH	0.41 at 10 mA/cm^2	-	OER	[20]
CoP-4ph-CMP-800	GCE	1 M KOH	0.43 at 10 mA/cm^2	-	OER	[20]
(CoP)n-MWCNTs [ae]	GCE	1 M KOH	0.29 at 1 mA/cm^2	0.055	OER	[21]
CoP-TIPS/MWCNTs [af]	GCE	1 M KOH	0.44 at 1 mA/cm^2	-	OER	[21]
[ERGO@CoTMPyP]$_7$/ PDDA/4-ABA/GC [ag]	GCE	0.1 M KOH	0.474 at –1 mA/cm^2	0.116	HER	[22]
$G_{ZnP\text{-}DMF\text{-}1}$	graphite	1 M KOH	0.52 at –10 mA/cm^2	0.15	HER	This work
$G_{CoP\text{-}DMSO\text{-}1}$	graphite	0.5 M H_2SO_4	0.51 at 10 mA/cm^2	0.27	OER	This work

[a] Zn-TPP = 5,10,15,20-tetraphenyl-21H,23H-porphine
[b] Zn-TAPP = 5,10,15,20-tetrakis(4-aminophenyl)-21H,23H-porphine
[c] Zn-TPyP = 5,10,15,20-tetrakis(4-pyridyl)-21H,23H-porphine
[d] ZnTAPP-NA = Zn(II) 5,10,15,20-tetra(4-aminophenyl)-21H,23H-porphyrin - ferrocene-1,1'-dicarbaldehyde
[e] GCE = glassy carbon electrode
[f] CoTAPP-NA = Co(II) 5,10,15,20-tetra(4-aminophenyl)-21H,23H-porphyrin - ferrocene-1,1'-dicarbaldehyde
[g] CoTcPP/ZrP = Co(II) meso-tetra(4-carboxyphenyl)porphyrin/zirconium phosphate
[h] GCRDE = glassy carbon rotating disk electrode

[i] $(Co–P)_{0.5}(Fe–P)_{0.5}$@CNT = hybrid formed by loading Co(II) tetrakis(pentafluorophenyl)porphyrin and Fe(III) chloride tetrakis(pentafluorophenyl)porphyrin on carbon nanotubes
[j] 3,4,5-OMe-CoP/CNT = Co(II) 5,10,15,20-tetra(3,4,5-trimethoxyphenyl)porphyrin / carbon nanotubes
[k] 2,4,6-OMe-CoP/CNT = Co(II) 5,10,15,20-tetra(2,4,6-trimethoxyphenyl)porphyrin / carbon nanotubes
[l] 1/CNTs = Co(II) tetra(phenyl)porphyrin / carbon nanotubes
[m] RDE = rotating ring-disk electrode
[n] 2/CNTs = Co(II) tetra(pentafluorophenyl)porphyrin / carbon nanotubes
[o] 1/MWCNT = Co(II) tetraphenylporphyrin immobilized on multi-walled carbon nanotubes
[p] 1/MWCNT/Py-Py = nanocomposite with pyrene-pyridine hybrid used as an axial ligand to bridge Co(II) tetraphenylporphyrin immobilized on multi-walled carbon nanotubes
[q] CoTPP-SD = Co(II) 5,10,15,20-tetrakis(4-aminophenyl)porphyrin – salicylaldehyde
[r] CFP = carbon fibre paper
[s] CoCOP = Co(II) 5,10,15,20-tetrakis(4-aminophenyl)porphyrin-based covalent organic polymer
[t] CoTCPP = Co(II) meso-tetra(4-carboxyphenyl)porphyrin
[u] CoTCPP polymer = crystalline Co(II) meso-tetra(4-carboxyphenyl)porphyrin-based polymeric system
[v] PIZA-1-400 = Co(II) 5,10,15,20-(4-carboxyphenyl)porphyrin network thin film calcined at 400 °C
[w] CeO_2@PIZA-1-400 = Co(II) 5,10,15,20-(4-carboxyphenyl)porphyrin network thin film with encapsulated CeO_2 and calcined at 400 °C
[x] CoTMPyP/ERGO = tetrakis(N-methylpyridyl)porphyrinato cobalt / electrochemically reduced graphene oxide
[y] Co-MPPy-1 = Co(II)-porphyrin / pyrene comprised conjugated microporous polymer
[z] Co-2DP = multilayer 2D polymer based on Co(II) 5,10,15,20-tetrakis(4-aminophenyl)-21H,23H-porphyrin and 2,5-dihydroxy-terephthalaldehyde
[aa] $rGO/(Ni^{2+}–THPP/Co^{2+}–THPP)_8$ = multilayer structure based on Ni(II) and Co(II) 5,10,15,20-tetrakis(4-hydroxyphenyl)porphyrins assembled on reduced graphene oxide sheets
[ab] CoP-2ph-CMP-800, [ac] CoP-3ph-CMP-800 and [ad] CoP-4ph-CMP-800 = conjugated mesoporous polymer based on Co-porphyrins and pyrolyzed at 800 ºC
[ae] (CoP)n-MWCNTs = multilayer covalent Co(II) 5,10,15,20-tetraethynyl-porphyrin framework on multi-walled carbon nanotubes
[af] CoP-TIPS/MWCNTs = Co(II) 5,10,15,20-tetrakis(triisopropylsilylethynyl)porphyrin mixed with multi-walled carbon nanotubes
[ag] $[ERGO@CoTMPyP]_7$/PDDA/4-ABA/GC = multilayer films containing tetrakis(N-methylpyridyl)porphyrinato cobalt, on treated glassy carbon electrode

References

[1] I. Fratilescu, A. Lascu, B.O. Taranu, C. Epuran, M. Birdeanu, A.-M. Macsim, E. Tanasa, E. Vasile, E. Fagadar-Cosma, One A_3B porphyrin structure—Three successful applications, Nanomater. 12 (2022) 1930;
[2] B.-O. Taranu, M.-G. Ivanovici, P. Svera, P. Vlazan, P. Sfirloaga, M. Poienar, Ni_{11}□$(HPO_3)_8(OH)_6$ multifunctional materials: Electrodes for oxygen evolution reaction and potential visible-light active photocatalysts, J. Alloys Compd. 848 (2020) 156595;
[3] M. Poienar, B.-O. Taranu, P. Svera, P. Sfirloaga, P. Vlazan, Disclosing the thermal behaviour, electrochemical and optical properties of synthetic $Fe_3(PO_4)_2(OH)_2$ materials, J. Therm. Anal. Calorim. (2022) 1-17 https://doi.org/10.1007/s10973-022-11435-z;
[4] S. Motoc, F. Manea, C. Orha, A. Pop, Enhanced electrochemical response of diclofenac at a fullerene–carbon nanofiber paste electrode, Sensors (Switzerland) 19 (2019) 1332;
[5] M. Yang, Y. Yang, Y. Liu, G. Shen, R. Yu, Platinum nanoparticles-doped sol-gel/carbon nanotubes composite electrochemical sensors and biosensors, Biosens. Bioelectron. 21 (2006) 1125–1131;
[6] S. Seo, K. Lee, M. Min, Y. Cho, M. Kim, H. Lee, A molecular approach to an electrocatalytic hydrogen evolution reaction on single-layer graphene, Nanoscale 9 (2017) 3969-3979;
[7] G. Cai, L. Zeng, L. He, S. Sun, Y. Tong, J. Zhang, Imine gels based on ferrocene and porphyrin and their electrocatalytic property, Chem Asian J. 15 (2020) 1963–1969;
[8] I.B. Alvarez, Y. Wu, J. Sanchez, Y. Ge, M.V. Ramos-Garces, T. Chu, T.F. Jaramillo, J.L. Colon, D. Villagran, Cobalt porphyrin intercalation into zirconium phosphate layers for electrochemical water oxidation, Sustain. Energy Fuels 5 (2021) 430-437;
[9] H. Lei, Q. Zhang, Y. Wang, Y. Gao, Y. Wang, Z. Liang, W. Zhang, R. Cao, Significantly boosted oxygen electrocatalysis with cooperation between cobalt and iron porphyrins, Dalton Trans. 50 (2021) 5120-5123;
[10] H. Lv, H. Guo, K. Guo, H. Lei, W. Zhang, H. Zheng, Z. Liang, R. Cao, Substituent position effect of Co porphyrin on oxygen electrocatalysis, Chin. Chem. Lett. 32 (2021) 2841–2845.
[11] H. Qin, Y. Wang, B. Wang, X. Duan, H. Lei, X. Zhang, H. Zheng, W. Zhang, R. Cao, Cobalt porphyrins supported on carbon nanotubes as model catalysts of metal-N_4/C sites for oxygen electrocatalysis, J. Energy Chem. 53 (2021) 77–81;
[12] I.K. Attatsi, W. Zhu, X. Liang, Noncovalent immobilization of Co(II)porphyrin through axial coordination as an enhanced electrocatalyst on carbon electrode for oxygen reductions and evolutions, New J. Chem. 44 (2020) 4340-4345;
[13] A. Wang, L. Cheng, W. Zhao, X. Shen, W. Zhu, Electrochemical hydrogen and oxygen evolution reactions from a cobalt-porphyrin-based covalent organic polymer, J. Colloid Interface Sci. 579 (2020) 598–606;

[14] Y. Wu, J.M. Veleta, D. Tang, A.D. Price, C.E. Botez, D. Villagran, Efficient electrocatalytic hydrogen gas evolution by a cobalt-porphyrin-based crystalline polymer, Dalton Trans. 47 (2018) 8801-8806;
[15] D.-J. Li, Z.-G. Gu, W. Zhang, Y. Kang, J. Zhang, Epitaxial encapsulation of homodispersed CeO_2 in a cobalt-porphyrin network derived thin film for highly efficient oxygen evolution reaction, J. Mater. Chem. A 5 (2017) 20126-20130;
[16] J. Ma, L. Liu, Q. Chen, M. Yang, D. Wang, Z. Tong, Z. Chen, A facile approach to prepare crumpled CoTMPyP/electrochemically reduced graphene oxide nanohybrid as an efficient electrocatalyst for hydrogen evolution reaction, Appl. Surf. Sci. 399 (2017) 535–541;
[17] S. Bhunia, K. Bhunia, B.C. Patra, S.K. Das, D. Pradhan, A. Bhaumik, A. Pradhan, S. Bhattacharya, Efficacious electrochemical oxygen evolution from novel Co(II) porphyrin-pyrene based conjugated microporous polymer, ACS Appl. Mater. Interfaces 11 (2019) 1520–1528;
[18] H. Sahabudeen, H. Qi, B.A. Glatz, D. Tranca, R. Dong, Y. Hou, T. Zhang, C. Kuttner, T. Lehnert, G. Seifert, U. Kaiser, A. Fery, Z. Zheng, X. Feng, Wafer-sized multifunctional polyimine-based two-dimensional conjugated polymers with high mechanical stiffness, Nat. Commun. 7 (2016) 13461;
[19] J. Sun, H. Yin, P. Liu, Y. Wang, X. Yao, Z. Tang, H. Zhao, Molecular engineering of Ni–/Co–porphyrin multilayers on reduced graphene oxide sheets as bifunctional catalysts for oxygen evolution and oxygen reduction reactions, Chem. Sci. 7 (2016) 5640-5646;
[20] H. Jia, Y. Yao, Y. Gao, D. Lu, P. Du, Pyrolyzed cobalt porphyrin-based conjugated mesoporous polymers as bifunctional catalysts for hydrogen production and oxygen evolution in water, Chem. Commun. 52 (2016) 13483-13486;
[21] H. Jia, Z. Sun, D. Jiang, P. Du, Covalent cobalt porphyrin framework on multi-walled carbon nanotubes for efficient water oxidation at low overpotential, Chem. Mater. 27 (2015) 4586–4593;
[22] D. Huang, J. Lu, S. Li, Y. Luo, C. Zhao, B. Hu, M. Wang, Y. Shen, Fabrication of cobalt porphyrin. Electrochemically reduced graphene oxide hybrid films for electrocatalytic hydrogen evolution in aqueous solution, Langmuir 30 (2014) 6990–6998.
